# New York College of Veterinary Surgeons

**Published:** 1896-09

**Authors:** 


### NEW YORK COLLEGE OF VETERINARY SURGEONS.

VERY many changes are noted in this school for the ensuing year. Willard A. Cobb, M.A., 1886, Lockport, N. Y., term expires on the Board of Regents; from the Board of Trustees C. E. Billington, M.D., retires, and is succeeded by William Anderson, V.S. Allen S. Heath, M.D.V.S., professor of breeding and management of horses and cattle, retires. August C. Hassloch, V.S., lecturer on materia medica and therapeutics, retires, and is succeeded by J. Mosedale, M.R.C.V.S., as instructor. Frederick T. Reyling, M.D., is succeeded by Alfred L. Beebe, Ph.B., as lecturer on bacteriology, and Ferdinand M. Jeffries, M.D., will fill the rôle of demonstrator in histology. The death of David Roberge, lecturer on horseshoeing, creates a vacancy which will be filled by P. Burns, V.S., as demonstrator. Thomas Giffen, M.R.C.V.S., has been added as lecturer on the external form of the horse and examination for soundness. Edwin Parsons, D.V.S., will be demonstrator in equine dentistry. Louis P. Cook, D.V.S., will assist the chair of anatomy. Joseph N. B. Rawle, M.D.V.S., will lecture on veterinary jurisprudence. George F. Horpel, V.S., has been added as assistant to the chair of clinical medicine. Edward N. Leavy, D.V.S., will lecture on zoötechnics. Samuel Goldstein will lecture on diseases of the throat and nose, a new departure in veterinary medicine. Joseph Thackaberry, V.S., succeeds M. O'M. Knott, V.S., as assistant demonstrator of anatomy. Rudolph J. Schrieber, Ph.G., V.S., retires as assistant in botany.

The demands for higher and broader work in New York State seem to be fully realized by the schools, and every effort to attain the most thorough work is entered upon with such zeal and determined action that every friend of a better education for the veterinarian must feel happy and proud of the results in store for those who are to follow in our footsteps. Such evidences of good intention merit from one and all the strongest support and the kindly hand of assistance at all times.

